# Octocrylene: From Sunscreens to the Degradation Pathway during Chlorination Processes: Formation of Byproducts and Their Ecotoxicity Assessment

**DOI:** 10.3390/molecules27165286

**Published:** 2022-08-19

**Authors:** Antonio Medici, Lorenzo Saviano, Antonietta Siciliano, Giovanni Libralato, Marco Guida, Lucio Previtera, Giovanni Di Fabio, Armando Zarrelli

**Affiliations:** 1 Department of Chemical Sciences, University of Naples Federico II, Via Vicinale Cupa Cintia 26, 80126 Naples, Italy; 2 Department of Biology, University of Naples Federico II, Via Vicinale Cupa Cintia 26, 80126 Naples, Italy; 3 Associazione Italiana per la Promozione delle Ricerche su Ambiente e Salute Umana, 82030 Dugenta, Italy

**Keywords:** octocrylene, chlorination, hypochlorite, degradation byproducts, water treatment, *Aliivibrio fischeri*, *Phaeodactylum tricornutum*, *Brachionus plicatilis*

## Abstract

Octocrylene is an organic sunscreen whose main action is to absorb UVB radiation and short UVA wavelengths; it is used in various cosmetic products in order to provide an adequate sun-protection factor or to protect the cosmetic formulations themselves from UV radiation. This filter is believed to be a possible endocrine disruptor and is also questioned due to its allergic and/or photoallergic potential. However, it continues to be widely used, and it has been found in various environments, not least those of swimming pools, where it is evidently released by consumers, to the point that it is now considered an emerging micropollutant. The present investigation presents the possible chemical fate of octocrylene in the typical chlorination conditions of wastewater or swimming pools. A total of 11 disinfection byproducts were identified, and 6 were identified for the first time, and separated by HPLC. These products were identified through careful mass spectrometry studies and 1D and 2D NMR experiments. A formation mechanism has been proposed that justifies the chemical structures of all of the compounds identified. The ecotoxicological assessment of octocrylene and their products was carried out by employing *Phaeodactylum tricornutum*, *Brachionus plicatilis* and *Aliivibrio fischeri* as bioindicators. The ecotoxicity results reveal that toxic byproducts might be generated during the oxidation process, increasing the potential risk to the marine environment.

## 1. Introduction

Octocrylene (OCT) is a viscous, clear and colorless oil, introduced in commercial sunscreens and anti-aging creams about 15 years ago. It is the 2-ethylhexyl ester of 2-cyano-3,3-diphenylacrylic acid, with the extended conjugation of the acid portion that absorbs UVB and short-wave UVA (ultraviolet) rays, with wavelengths from 280 to 320 nm [1], which promote tanning but also contribute to the onset of sunburn and skin cancer. It is used in various body care products [2,3], in concentrations up to 10%, in order to provide an adequate sun-protection factor or to protect the body care formulations themselves from UV radiation. This filter was recently indicted for the risk of inducing potential adverse effects on the endocrine system [4], as well as having an allergic and/or photoallergic potential [5]. In recent years, there has been an increase in the number of cases of photocontact allergic reactions to octocrylene, which has been referred to as an “emerging allergen”. However, it has the advantage of working in synergy, allowing for wide and beneficial photoprotection; for example, it stabilizes avobenzone (butyl methoxydibenzoylmethane), a molecule present in the UVA filter. The European Chemicals Agency (ECHA) constantly evaluates the safety profile of this filter, like all chemicals used in cosmetics and registered under European legislation. Octocrylene has been found in various environments, not least those of swimming pools [6], where it is evidently released by consumers, to the point that it is now considered an emerging micropollutant similar to polyfluoroalkyl substances (PFAS) [7], blue-green algae [8], toxic fungal products [9], hormones [10], psychoactive drugs [11,12], pesticides [13], cosmetics, and industrial additives and drugs [14,15,16,17,18]. These substances, unlike conventional and unconventional pollutants, are still largely unregulated by legislation and are not restricted by maximum permitted values. Furthermore, they are potentially dangerous for the environment and human health, even in an overall context of insufficient data linked to their dangerousness [19,20,21,22,23]. Removal of emerging contaminants from wastewater can be accomplished by ozonation [24], membrane filtration [25], adsorption [26], and, above all, advanced oxidation [27,28].

In this paper, the degradation byproducts (DPs) of OCT were investigated by mimicking the chlorination process normally used in swimming pools to sterilize and disinfect water and to reduce similar emerging pollutants [6,29]. In particular, two different experiments were carried out, one at concentrations of about 10^−5^ M, comparable to those at which OCT is present in wastewater, and one at concentrations at least 100 times higher in order to isolate and identify the DPs. The structures of 11 isolated DPs, 6 of which were isolated for the first time, were determined by crossing the data provided by nuclear magnetic resonance (NMR) and those obtained by mass spectrometry (MS), using matrix-assisted laser desorption/ionization as a source and a time-of-flight analyzer (MALDI-TOF) for mass spectroscopy. It was also possible to propose a mechanism of formation that justifies the obtainment of the isolated products.

The spatial distribution of OCT is strictly connected to its presence in the marine aquatic environment; this presence is due to the anthropogenic activities responsible for its direct emission. For this reason, an ecotoxicological assessment was carried out with marine aquatic bioindicators such as *Aliivibrio fischeri*, *Phaeodactylum tricornutum* and *Brachionus plicatilis*. Emerging organic pollutants such as UV filters therefore require biological assays capable of detecting potential toxicity from a one-health perspective.

## 2. Results and Discussion

### 2.1. Chlorination Experiments

The OCT chlorination experiments were performed in the concentrations in which this micropollutant was detected in swimming pool water [6], of approximately 10^−5^ M. Specifically, the solutions of the sunscreen were treated for 10 min with 10% hypochlorite (OCT: hypochlorite molar ratio of 2:1; concn.), under magnetic stirring and at room temperature. Then, the tests were repeated at much higher concentrations of the contaminant (>10^−3^ M), with a much lower ratio of OCT: oxidizing agent (1:20), in order to have sufficiently high quantities of byproducts isolated to proceed with the structural identification.

**Scheme 1:**
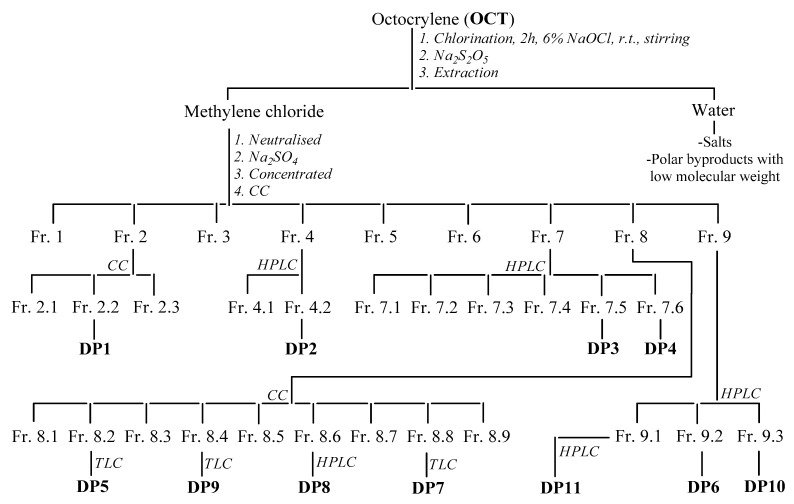
The isolation of the degradation byproducts.

**Figure 1:**
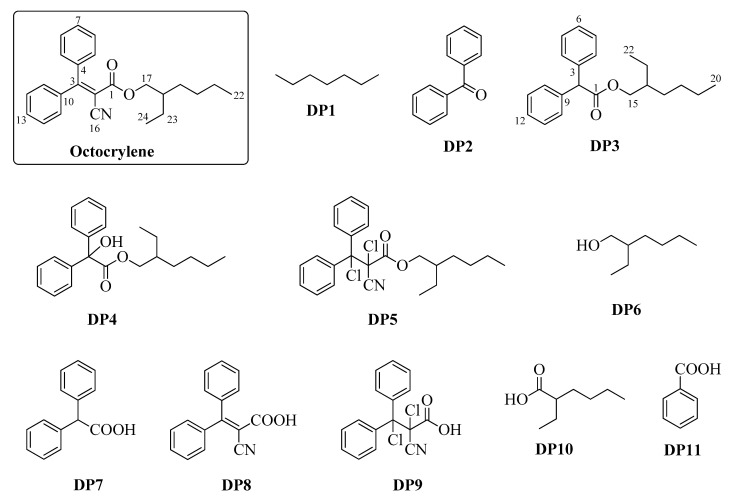
Chemical structures of octocrylene and its degradation byproducts **DP1**–**DP11**.

**Figure 2:**
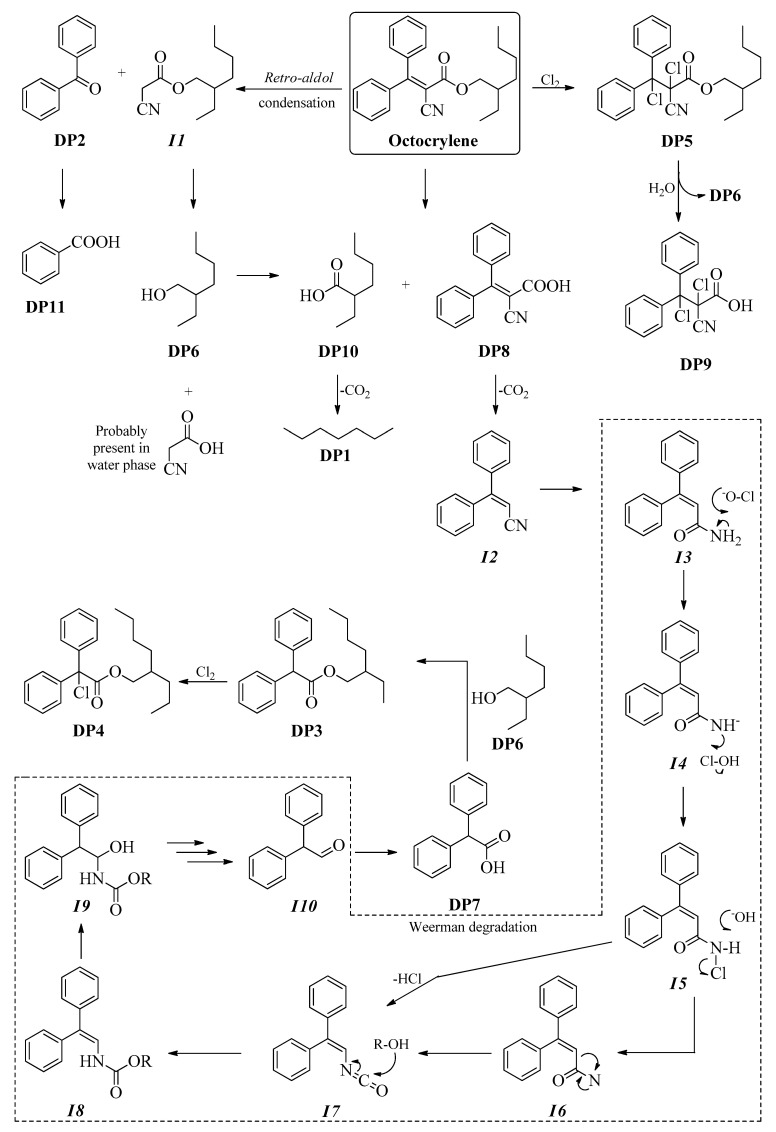
Plausible mechanism for the formation of **DP1**-**DP11**.

The course of the reaction was monitored by HPLC, and the DPs obtained were isolated according to Scheme 1, using column chromatography and HPLC and completely characterized using NMR and MS analyses. Finally, **DP1**–**DP11** (Figure 1) were isolated at percentages of 1.12, 2.97, 0.89, 0.91, 1.15, 2.36, 1.08, 1.55, 6.39, 0.59, and 1.19, respectively. The proposed mechanism of their formation from OCT is shown in Figure 2, and **DP3**–**DP5** and **DP7**–**DP9** were isolated for the first time.

### 2.2. Structure Elucidation of Degradation Byproducts DP1–DP9

In the OCT treatment, the concentration of **DP1**–**DP11** reached its maximum after about 2 h, with a degradation of 15% and a transformation of approximately 20%; percentages of byproducts ranged from 0.59% for **DP10** to 6.39% for **DP9**. In a basic environment, OCT can undergo a retro-aldol condensation, which leads to the formation of intermediate *I1* and the byproduct **DP2**. From this and other byproducts, it is usually possible to obtain the byproduct **DP11**; the intermediate *I1*, identifiable with 2-ethylhexyl cyanoacetate, can undergo hydrolysis of the ester bond and lead to the formation of the byproduct **DP10** and α-cyanoacetic acid, probably contained in the aqueous phase rich in salts and low-molecular-weight compounds (Scheme 1). Finally, the **DP10** could decarboxylate to the **DP1** byproduct. The byproduct **DP10** is also obtained from the hydrolysis of the ester bond of the starting product, together with the byproduct **DP8**. The latter, by decarboxylation, could provide the intermediate *I2*, which, by the hydrolysis of the cyano group, provides the intermediate *I3*. Considering the degradation reaction of OCT in the presence of sodium hypochlorite, it can be assumed that a Weerman degradation takes place that leads to the formation of nitrene *I6*, through the deprotonation (*I4*) and chlorination (*I5*) of the amide nitrogen of the intermediate *I3*.

The transposition of nitrene *I6*, or more probably the elimination of HCl from the intermediate *I5* with the concomitant transposition of the residue bound to the carbonyl, allows for the obtainment of the isocyanate *I7*, from which alcoholises of the intermediate *I8* are obtained, from which, for the subsequent oxidation, the intermediate *I9* is obtained. The hydrolysis of the latter and the subsequent oxidation of the intermediate *I10* obtained provides the byproduct **DP7**. This can react with the **DP6** present in the solution and provide the byproduct **DP3** and, for subsequent chlorination, create the byproduct **DP4**. The direct chlorination to the C-2/C-3 carbons of the starting product allows for and explains the obtainment of the byproduct **DP5**, from which **DP9** and **DP6** are obtained by the hydrolysis of the ester bond.

### 2.3. Spectral Data

Octocrylene: *2-Ethylhexyl 2-cyano-3,3-diphenylacrylate*. Oily liquid. ^1^H- and ^13^C-NMR, see Table S1. MS-TOF (positive ions): *m/z* calculated for C_24_H_27_NO_2_ *m/z* 361.20 [M]^+^; found 362.47 [M + H]^+^ (68%).

**DP1**: *Heptane*. Identified by comparison with an authentic sample.

**DP2**: *Benzophenone*. Identified by comparison with an authentic sample.

**DP3**: *2-Ethylhexyl 2,2-diphenylacetate*. White powder. ^1^H- and ^13^C-NMR, see Table S2. MS-TOF (positive ions): *m/z* calculated for C_22_H_28_O_2_ *m/z* 324.21 [M]^+^; found 325.47 [M + H]^+^.

**DP4**: *2-Ethylhexyl 2-chloro-2,2-diphenylacetate*. White powder. ^1^H- and ^13^C-NMR, see Table S3. MS-TOF (positive ions): *m/z* calculated for C_22_H_27_ClO_2_ *m/z* 358.17 [M]^+^; found 361.82 [M + H]^+^, 359.78 [M + H]^+^.

**DP5**: *(2S)-2-Ethylhexyl 2,3-dichloro-2-cyano-3,3-diphenylpropanoate*. White powder. ^1^H- and ^13^C-NMR, see Table S4. MS-TOF (positive ions): *m/z* calculated for C_24_H_27_Cl_2_NO_2_ *m/z* 431.14 [M]^+^; found 436.33 [M + H]^+^, 435.36 [M + H]^+^, 434.38 [M + H]^+^, 433.35 [M + H]^+^, 432.36 [M + H]^+^.

**DP6**: *2-Ethylhexan-1-ol*. Identified by comparison with an authentic sample.

**DP7**: *2,2-Diphenylacetic acid*. White powder. ^1^H- and ^13^C-NMR, see Table S5. MS-TOF (positive ions): *m/z* calculated for C_14_H_12_O_2_ *m/z* 212.08 [M]^+^; found 213.27 [M + H]^+^.

**DP8**: *2-Cyano-3,3-diphenylacrylic acid*. White powder. ^1^H- and ^13^C-NMR, see Table S6. MS-TOF (positive ions): *m/z* calculated for C_16_H_11_NO_2_ *m/z* 249.08 [M]^+^; found 250.19 [M + H]^+^.

**DP9**: *(S)-2,3-dichloro-2-cyano-3,3-diphenylpropanoic acid*. White powder. ^1^H- and ^13^C-NMR, see Table S7. MS-TOF (positive ions): *m/z* calculated for C_16_H_11_Cl_2_NO_2_ *m/z* 319.02 [M]^+^; 324.18 [M + H]^+^, 323.16 [M + H]^+^, 322.19 [M + H]^+^, 321.18 [M + H]^+^, 320.21 [M + H]^+^.

**DP10**: *2-ethylhexanoic acid*. Identified by comparison with an authentic sample.

**DP11**: *Benzoic acid*. Identified by comparison with an authentic sample.

### 2.4. Toxicity Assessment

Octocrylene has a relatively high environmental stability in aquatic environments and is hardly removed from wastewater treatment plants [30,31]. Previous studies showed that OCT was poorly removed from wastewater treatment plants (0–10% degradation in aerobic conditions) [32].

**Figure 3:**
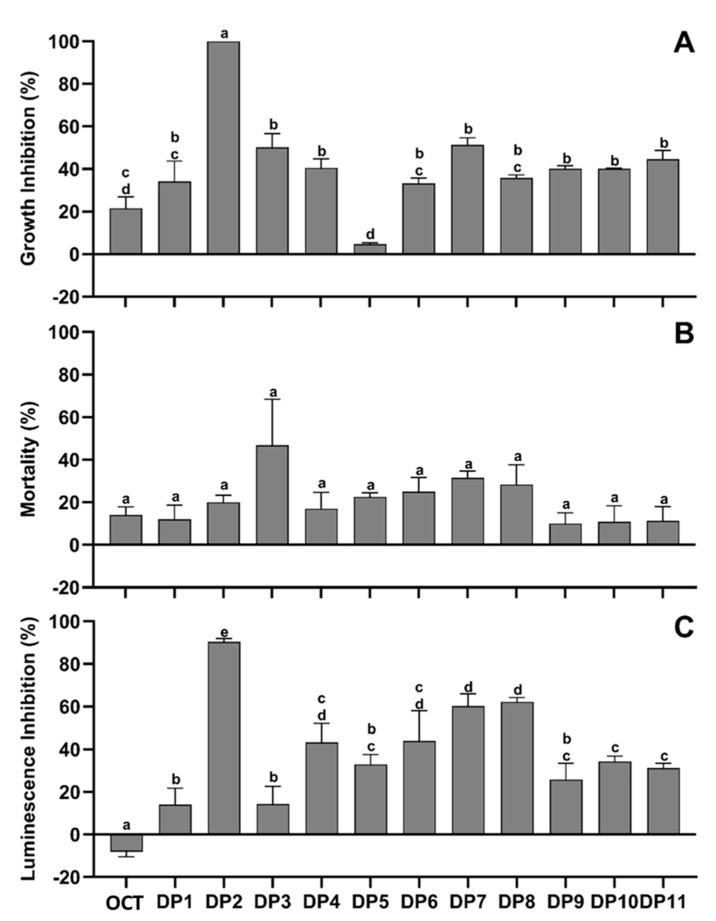
Toxicity data regarding exposure of *P. tricornutum* (**A**), *B. plicatilis* (**B**) and *A. fischeri* (**C**) to OCT and its byproducts (**DP1**-**DP11**). Data with different letters (a–d) are significantly different (Tukey post hoc, *p* < 0.05).

Toxicity data were reported in Figure 3A–C for *P. tricornutum*, *B. plicatilis* and *A. fischeri*, in that order, considering the effect of OCT and its byproducts.

Chronic toxicity with *P. tricornutum* was also identified, as 83% of DPs showed growth inhibition effects ranging between 20% and 50% (OCT, **DP1**, **DP3**, **DP4**, **DP6**, **DP7**, **DP8**, **DP9**, **DP10** and **DP11**). **DP5** has no toxicity, while **DP2** was the most toxic compound (Figure 3A).

The effects of this UV filter and its chlorinated derivates on the acute toxicity of *B. plicatilis* change, probably due to the lower sensibility of the bioindicator. The toxicity has been evaluated by observing the mortality rate of *B. plicatilis* after 24 h of exposure. As reported in Figure 3B, no significant effect on mortality was observed in rotifers exposed to OCT and its degradation byproducts. In fact, all the investigated samples showed a toxicity ranging between 10% and 32%, with the exception of **DP3,** which had a residual toxicity of approximately 47%. In the latter case study, **DP2**, on the other hand, has a toxicity of only 20%, while **DP3** appears to be the most toxic degradation byproduct for the aforementioned bioindicator.

The acute toxicity of OCT and its chlorinated derivatives towards *A. fischeri* was shown in Figure 3C. After 30 min of exposure, the bioluminescence inhibition swung between 14% and 90%, except for the parent compound OCT, which was the only compound to exhibit biostimulation behaviour. So, 16% of the tested samples did not exceed 20% of the effect (**DP1** and **DP3**), 33% of the degradation byproducts are included in the range between 26% and 33% of the effect (**DP5**, **DP9**, **DP10** and **DP11**), while there was another 33% increase in toxicity up to 60% of the effect (**DP4**, **DP6**, **DP7** and **DP8**). **DP2** (90% of the effect), once again, appears to be the most toxic product. The toxicity trend observed in *A. fischeri* is in good agreement with our previous results on *P. tricornutum*.

The discharge into the marine environment of chlorinated sewage effluents containing these degradation byproducts represents the worst-case scenario for environmental safety; indeed, in our study, these byproducts could have negatively influenced the physiology of single-celled organisms such as *A. fischeri* and *P. tricornutum*, but they did not affect rotifers. This study highlighted the concerns and the potential risks from OCT byproducts that may emerge and impact the quality of the marine ecosystem, especially concerning uncontrolled doses.

## 3. Materials and Methods

### 3.1. Drug and Reagents

Octocrylene (99%) was purchased from Sigma Aldrich (Milan, Italy). All of the other chemicals and solvents were purchased from Sigma Aldrich (Milan, Italy) and were of HPLC grade and used as received. All of the chemicals were of analytical grade and supplied by Sigma Aldrich.

The toxicity tests were conducted with two combinations. In the first combination, osmotic adjustment solution (OAS) (22 g L^−1^ NaCl) was used as a control for optimal conditions according to the ISO 11348-3 standard [33]. In the second combination, synthetic sea water was used as the control solution according to the ISO 10,253 standard [34,35]. The synthetic sea water used for analytical procedures comprised the following salts: NaCl (22 g L^−1^), MgCl_2_·6H_2_O (9.7 g L^−1^), Na_2_SO_4_ (3.7 g L^−1^), CaCl_2_ (1.0 g L^−1^), KCl (0.65 g L^−1^), NaHCO_3_ (0.2 g L^−1^) and H_3_BO_3_ (0.023 g L^−1^).

### 3.2. Chlorination Reaction

#### 3.2.1. Apparatus and Equipment

Column chromatography (CC) was carried out with Kieselgel 60 (230–400 mesh, Merck, Darmstadt, Germany). HPLC was performed on a Shimadzu LC-8A system using a Shimadzu SPD-10A VP UV-VIS detector (Shimadzu, Milan, Italy). Preparative HPLC was performed using an RP Gemini C18-110A preparative column (10 μm particle size, 250 mm × 21.20 mm i.d. Phenomenex, Bologna, Italy) with a flow rate of 8.0 mL/min. The ^1^H- and ^13^C-NMR spectra were recorded with an NMR spectrometer operated at 400 MHz and at 25 °C (Bruker DRX, Bruker Avance) and referenced in ppm to the residual solvent signals (CDCl_3_, at δ_H_ 7.27 and δ_C_ 77.0; and CD_3_OD, at δ_H_ 3.30 and δ_C_ 49.0). The proton-detected heteronuclear correlations were measured using a gradient heteronuclear single-quantum coherence (HSQC) experiment, optimized for ^1^J_HC_ = 155 Hz, and a gradient heteronuclear multiple bond coherence (HMBC) experiment, optimized for ^n^J_HC_ = 8 Hz. The MALDI-TOF mass spectrometric analyses were performed on a Voyager-De Pro MALDI mass-spectrometer (PerSeptive Biosystems, Framingham, MA, USA). The samples were lyophilized using a Lyovapor^TM^-200 (Buchi, Cornaredo (MI), Italy), with a compressor with cooling capacity: 1.97 kW for 50 Hz and minimum condenser temperature: −55 °C.

#### 3.2.2. Chlorination Experiments

A 10^−5^ M OCT solution was treated for 10 min with 10% hypochlorite (molar ratio OCT/HClO 2:1 concentration, spectroscopically determined λ_max_ 292 nm, ε 350 dm^3^/mol cm) at room temperature [36]. The presence of OCT was quantified using a Lambda 12 UV-Vis spectrophotometer (Perkin Elmer, USA). Absorbance peaks were determined at 310 nm. The absorbance values were converted into a concentration using a calibration curve prepared from standard solutions with known OCT concentrations. **DP1**–**DP11** were isolated from the methylene chloride extract of the aqueous solution (Scheme 1 and Figure 1) and identified by comparing their retention times with those of commercially available standard compounds, or isolated by performing preparative experiments with a solution of OCT at a concentration higher than 10^−3^ M and treated with 6% hypochlorite at room temperature for 2 h. The pH of the solution, measured and recorded continuously using a pH-meter, increased immediately from the initial pH of 8.0 to 10.8, and the pH remained at this value during the reaction. An aliquot of the solution was taken every 15 min, quenched by sodium thiosulphate excess, filtered, and dried by lyophilisation, and the residue was dissolved in a saturated sodium bicarbonate solution and extracted with ethyl acetate. The course of the reaction was monitored using HPLC. The DPs obtained were isolated using CC and HPLC and were completely characterized using NMR and MS analyses.

#### 3.2.3. Chlorination Procedure and Product Isolation

Octocrylene (607 mg, 1.68 mmol) was dissolved in 22 mL of acetonitrile, and the solution was diluted with water until a final volume of 0.9 L was reached. A sodium hypochlorite solution (approximately 6% active chlorine, molar ratio OCT/HClO 1:20; concentration spectroscopically determined at λ_max_ of 292 nm, ε = 350 dm^3^/mol cm) was added drop by drop to this solution under magnetic stirring at room temperature. The reaction was stopped after 2 h with an excess of sodium thiosulphate and concentrated by lyophilisation. The residue was dissolved in water and pH-adjusted to 5.0, and this solution was extracted using methylene chloride. The crude organic fraction (835 mg) was chromatographed on silica gel CC, eluted with a gradient of chloroform:methanol (99:1 to 10:90, *v*/*v*) to yield 9 fractions. The fraction Fr. 2 (62 mg), eluted with chloroform:methanol (97:3), was chromatographed on silica gel CC, eluted with a gradient of petroleum ether:acetone (98:2 to 90:10, *v*/*v*) to yield **DP1** (27 mg). The fraction Fr. 4 (49 mg), eluted with chloroform:methanol (90:10), was chromatographed on silica gel CC, eluted with a gradient of petroleum ether:acetone (90:10 to 50:50, *v*/*v*) to yield **DP2** (29 mg). The fraction Fr. 7 (58 mg), eluted with chloroform:methanol (60:40), was separated by semipreparative HPLC using a reversed-phase column Phenomenex Gemini 10 µm 110 Å C18 (250 × 21.20 mm) and eluted with a gradient of CH_3_COONH_4_ (A, pH 4.0; 10 mM) and methanol (B), starting with 30% B for 5 min and followed by the installation of a gradient to obtain 100% B over 30 min, at a solvent flow rate of 8 mL/min to yield **DP3** (7 mg) and **DP4** (3 mg). The fraction Fr. 8 (405 mg), eluted with chloroform:methanol (80:20), was chromatographed on silica gel CC, eluted with a gradient of methylene chloride:methanol (90:10 to 0:100, *v*/*v*) to yield 9 fractions.

The fraction Fr. 8.2 (61 mg), eluted with methylene chloride:methanol (90:10), was chromatographed on TLC, eluted with chloroform:methanol (80:20), to yield **DP5** (47 mg). The fraction Fr. 8.4 (131 mg), eluted with methylene chloride:methanol (80:20), was chromatographed on TLC, eluted with chloroform:methanol (70:30), to yield **DP9** (89 mg). The fraction Fr. 8.6 (43 mg), eluted with methylene chloride:methanol (65:35), was separated by analytical HPLC using a reversed-phase column Phenomenex Gemini 10 µm 110 Å C18 (250 × 21.20 mm) and eluted with a gradient of CH_3_COONH_4_ (A, pH 4.0; 10 mM) and methanol (B), starting with 30% B for 5 min and followed by the installation of a gradient to obtain 100% B over 30 min, and eluted again with the same mixture for another 10 min, at a solvent flow rate of 8 mL/min to yield **DP8** (20 mg). The fraction Fr. 8.8 (53 mg), eluted with methylene chloride:methanol (60:40), was chromatographed on TLC and eluted with petroleum ether:acetone (65:35), to yield **DP7** (20 mg).

The fraction Fr. 9 (31 mg), eluted with chloroform:methanol (90:10), was separated by analytical HPLC using a reversed-phase column Phenomenex Kromasil 10 µm 100 Å C18 (250 × 10.00 mm) and eluted with a gradient of CH_3_COONH_4_ (A, pH 4.0; 10 mM) and acetonitrile (B), starting with 20% B for 5 min and followed by the installation of a gradient to obtain 100% B over 30 min, at a solvent flow rate of 4 mL/min to give 3 fractions. The fraction 9.1 was purified by HPLC using a column Discovery RP-amide C16 (150 × 4.6 mm), 5 µm, and eluted with 0.1% TFA in acetonitrile:water (25:75), at a solvent flow rate of 0.8 mL/min to give **DP11** (2 mg). The fractions Fr. 9.2 and Fr. 9.3 were identified as **DP6** (5 mg) and **DP10** (2 mg), respectively.

### 3.3. Ecotoxicity Data

The toxicity of OCT and its degradation byproducts was assessed regarding the following organisms: *A. fischeri*, *P. tricornutum* and *B. plicatilis.* A Microtox^®^ acute ecotoxicity test was performed using the marine bioluminescent bacteria *A. fischeri* (NRRL-B-11177) to assess the toxicity of OCT and DPs. The bacteria were supplied in a freeze-dried form by Aqua Science LLC (Newark, Delaware, USA) and were stored at −20 °C to preserve their microbial activity. The acute toxicity endpoint was determined after 30 min of exposure according to ISO 11348-3 [33].

An algal growth-inhibition test was performed using benthic diatom *P. tricornutum*. The algal culture was kept at 20 ± 2 °C and 6000–10,000 Lux light, to obtain a cellular density of 10^6^ cells/mL. Inocula were taken from pre-cultures set up three days before the experiment to adjust the initial cell density to approximately 10^4^ cells/mL [35]. The test was carried out and miniaturized for 24-well sterile polystyrene micro-plates. The growth inhibition rate was calculated after 72 h exposure using a UV–Vis spectrophotometer (Hach Lange DR5000) and a 5 cm cuvette.

The acute toxicity test with estuarine rotifer *B. plicatilis* was performed according to the standard procedure of Rotoxkit M^®^ using certified dehydrated cysts (MicroBioTests Inc.). The test was conducted in multiwell plates with 300 μL per well. Six wells with ten rotifers each were filled to assess the toxicity of the parent compound and of its 11 DPs. Incubation was carried out for 48 h, at 25 °C, in darkness. The number of dead rotifers after the exposure period was observed under a stereomicroscope (LEICA EZ4-HD). The significance of the differences between the mean values of the different tests and controls was verified using Addinsoft XLSTAT (2016.02.27444 Version) by analysis of variance (ANOVA) with a 0.05 significance level. In addition, the post-hoc analyses were carried out with Tukey’s test.

## 4. Conclusions

This paper investigated the fate, following degradation treatment by chlorination, of one of the most widely used sunscreens, namely, octocrylene, conventionally considered an emerging micropollutant. The reaction was carried out by simulating the disinfection treatment employed in swimming pool waters, using excess sodium hypochlorite. After the chlorination treatment, chromatographic techniques were used to isolate eleven degradation byproducts, which were fully characterized by MS and NMR analyses and via comparison with a commercial standard. Four of them were isolated for the first time. Compared to the initial quantity considered, OCT was recovered unchanged for 45% and transformed into the corresponding byproducts for 20%. A possible mechanism for the degradation of OCT and its degradation byproducts has been hypothesized. Half of the investigated DPs possessed anywhere from slightly to highly toxic effects. Thus, acute toxicity evaluation demonstrated that the presence of OCT in the water distribution system might pose a more significant threat to safety and quality of the water and the environment. In fact, if on the one hand the disinfection process involves the partial degradation of OCT, it is also true, however, that it involves the formation of degradation byproducts, which are in some cases even more toxic than the starting product.

## Data Availability

Not applicable.

## Supplementary Materials

The following supporting information can be downloaded at: https://www.mdpi.com/article/10.3390/molecules27165286/s1, Table S1: ^1^H, ^13^C and 2D NMR data of octocrylene in CDCl_3_; Table S2: ^1^H, ^13^C and 2D NMR data of **DP2** in CDCl_3_; Table S3: ^1^H, ^13^C and 2D NMR data of **DP3** in CDCl_3_; Table S4: ^1^H, ^13^C and 2D NMR data of **DP4** in CDCl_3_; Table S5: ^1^H, ^13^C and 2D NMR data of **DP5** in CDCl_3_; Table S6: ^1^H, ^13^C and 2D NMR data of **DP7** in CDCl_3_; Table S7: ^1^H, ^13^C and 2D NMR data of **DP8** in CDCl_3_; Table S8: ^1^H, ^13^C and 2D NMR data of **DP9** in CDCl_3_.

## Sample Availability

Samples of the compounds are not available from the authors.

## References

[1] Berardesca E., Zuberbier T., Sanchez Viera M., Marinovich M. (2019). Review of the safety of octocrylene used as an ultraviolet filter in cosmetics. J. Eur. Acad. Dermatol. Venereol..

[2] de Groot A.C., Roberts D.W. (2014). Contact and photocontact allergy to octocrylene: A review. Contact Derm..

[3] Bennàssar A., Grimalt R., Romaguera C., Vilaplana J. (2009). Two cases of photocontact allergy to the new sun filter octocrylene. Dermatol. Online J..

[4] Carrotte-Lefebvre I., Bonnevalle A., Segard M., Delaporte E., Thomas P. (2003). Contact allergy to octocrylene: First 2 cases. Contact Derm..

[5] Karlsson I., Vanden Broecke K., Martensson J., Goossens A., Börje A. (2011). Clinical and experimental studies of octocrylene’s allergenic potency. Contact Derm..

[6] Mokh S., Nassar R., Berry A., Khatib M.E., Doumiati S., Taha M., Ezzeddine R., Al Iskandarani M. (2022). Chromatographic methods for the determination of a broad spectrum of UV filters in swimming pool water. Environ. Sci. Pollut. Res..

[7] Nakayama S.F., Yoshikane M., Onoda Y., Nishihama Y., Iwai-Shimada M., Takagi M., Kobayashi Y., Isobe T. (2019). Worldwide trends in tracing poly-and perfluoroalkyl substances (PFAS) in the environment. TRAC-Trend. Anal. Chem..

[8] Cheung M.Y., Liang S., Lee J. (2013). Toxin-producing cyanobacteria in freshwater: A review of the problems, impact on drinking water safety, and efforts for protecting public health. J. Microbiol..

[9] Kahn G., Vercellotti J.R. (2008). Abstracts of Papers of the American Chemical Society. CARB 100-Commercial Applications of Powdered Activated Carbons for Decolorizing Food Products such as Fruit Juice Concentrates and Sugar.

[10] Vieira W.T., de Farias M.B., Spaolonzi M.P., da Silva M.G.C., Vieira M.G.A. (2021). Endocrine-disrupting compounds: Occurrence, detection methods, effects and promising treatment pathways—A critical review. J. Environ. Chem. Eng..

[11] Valdez-Carrillo M., Abrell L., Ramírez-Hernández J., Reyes-López J.A., Carreón-Diazconti C. (2020). Pharmaceuticals as emerging contaminants in the aquatic environment of Latin America: A review. Environ. Sci. Pollut. Res..

[12] Romanucci V., Siciliano A., Galdiero E., Guida M., Luongo G., Liguori R., Di Fabio G., Previtera L., Zarrelli A. (2019). Disinfection by-products and Ecotoxic risk associated with hypochlorite treatment of tramadol. Molecules.

[13] Snow D.D., Cassada D.A., Bartelt–Hunt S.L., Li X., Zhang Y., Zhang Y., Yuan Q., Sallach J.B. (2012). Detection, occurrence and fate of emerging contaminants in agricultural environments. Water Environ. Res..

[14] Zarrelli A., DellaGreca M., Iesce M.R., Lavorgna M., Temussi F., Schiavone L., Criscuolo E., Parrella A., Previtera L., Isidori M. (2014). Ecotoxicological evaluation of caffeine and its derivatives from a simulated chlorination step. Sci. Total Environ..

[15] de Oliveira M., Frihling B.E.F., Velasques J., Magalhães Filho F.J.C., Cavalheri P.S., Migliolo L. (2020). Pharmaceuticals residues and xenobiotics contaminants: Occurrence, analytical techniques and sustainable alternatives for wastewater treatment. Sci. Total Environ..

[16] Romanucci V., Siciliano A., Guida M., Libralato G., Saviano L., Luongo G., Previtera L., Di Fabio G., Zarrelli A. (2020). Disinfection by-products and ecotoxic risk associated with hypochlorite treatment of irbesartan. Sci. Total Environ..

[17] Li P., Wu Y., He Y., Zhang B., Huang Y., Yuan Q., Chen Y. (2020). Occurrence and fate of antibiotic residues and antibiotic resistance genes in a reservoir with ecological purification facilities for drinking water sources. Sci. Total Environ..

[18] Jean J., Perrodin Y., Pivot C., Trepo D., Perraud M., Droguet J., Tissot-Guerraz F., Locher F. (2012). Identification and prioritization of bioaccumulable pharmaceutical substances discharged in hospital effluents. J. Environ. Manag..

[19] Mills L.J., Chichester C. (2005). Review of evidence: Are endocrine-disrupting chemicals in the aquatic environment impacting fish populations?. Sci. Total Environ..

[20] Zhou Y., Niu L., Zhu S., Lu H., Liu W. (2017). Occurrence, abundance, and distribution of sulfonamide and tetracycline resistance genes in agricultural soils across China. Sci. Total Environ..

[21] Zarrelli A., DellaGreca M., Parolisi A., Iesce M.R., Cermola F., Temussi F., Isidori M., Lavorgna M., Passananti M., Previtera L. (2012). Chemical fate and genotoxic risk associated with hypochlorite treatment of nicotine. Sci. Total Environ..

[22] Patel M., Kumar R., Kishor K., Mlsna T., Pittman Jr C.U., Mohan D. (2019). Pharmaceuticals of emerging concern in aquatic systems: Chemistry, occurrence, effects, and removal methods. Chem. Rev..

[23] Rivera-Utrilla J., Sánchez-Polo M., Ferro-García M.Á., Prados-Joya G., Ocampo-Pérez R. (2013). Pharmaceuticals as emerging contaminants and their removal from water. A review. Chemosphere.

[24] Ibáñez M., Gracia-Lor E., Bijlsma L., Morales E., Pastor L., Hernández F. (2013). Removal of emerging contaminants in sewage water subjected to advanced oxidation with ozone. J. Hazard. Mater..

[25] Kim S., Chu K.H., Al-Hamadani Y.A., Park C.M., Jang M., Kim D.-H., Yu M., Heo J., Yoon Y. (2018). Removal of contaminants of emerging concern by membranes in water and wastewater: A review. Chem. Eng. J..

[26] Yaghmaeian K., Moussavi G., Alahabadi A. (2014). Removal of amoxicillin from contaminated water using NH_4_Cl-activated carbon: Continuous flow fixed-bed adsorption and catalytic ozonation regeneration. Chem. Eng. J..

[27] Kanakaraju D., Glass B.D., Oelgemöller M. (2018). Advanced oxidation process-mediated removal of pharmaceuticals from water: A review. J. Environ. Manage..

[28] Luongo G., Previtera L., Ladhari A., Di Fabio G., Zarrelli A. (2020). Peracetic acid vs. sodium hypochlorite: Degradation and transformation of drugs in wastewater. Molecules.

[29] Teo T.L., Coleman H.M., Khan S.J. (2015). Chemical contaminants in swimming pools: Occurrence, implications and control. Environ. Int..

[30] Brausch J.M., Rand G.M. (2011). A review of personal care products in the aquatic environment: Environmental concentrations and toxicity. Chemosphere.

[31] Gago-Ferrero P., Alonso M.B., Bertozzi C.P., Marigo J., Barbosa L.R., Cremer M., Secchi E.R., Azevedo A., Lailson-Brito J., Torres J.P. (2013). First determination of UV filters in marine mammals. Octocrylene levels in Franciscana dolphins. Environ. Sci. Technol..

[32] Duis K., Junker T., Coors A. (2022). Review of the environmental fate and effects of two UV filter substances used in cosmetic products. Sci. Total Environ..

[33] (2007). Water quality-Determination of the Inhibitory Effect of Water Samples on the Light Emission of Vibrio Fischeri (Luminescent bacteria Test)-Part 3: Method Using Freeze-Dried Bacteria.

[34] Italian Parliament. Law 9 December 1998 n. 426. New Interventions in the Environmental Field.

[35] (2016). Water Quality—Marine Algal Growth Inhibition Test with Skeletonema sp. and Phaeodactylum Tricornutum.

[36] Bedner M., MacCrehan W.A. (2006). Transformation of acetaminophen by chlorination produces the toxicants 1, 4-benzoquinone and N-acetyl-p-benzoquinone imine. Environ. Sci. Technol..

