# Supplementary material for: Octocrylene: From Sunscreens to the Degradation Pathway during Chlorination Processes: Formation of Byproducts and Their Ecotoxicity Assessment
Source: Molecules. 2022 Aug 19;27(16):5286. doi: 10.3390/molecules27165286 (PMC9415856; doi:10.3390/molecules27165286)
Supplement: Supplementary file 1 [file molecules-27-05286-s001.zip › molecules-1854053-supplementary.pdf]

Supplementary Material

Octocrylene: from Sunscreens to the Degradation Pathway During Chlorination Processes: Formation of Byproducts and Their Ecotoxicity Assessment

Antonio Medici, Lorenzo Saviano, Antonietta Siciliano, Giovanni Libralato, Marco Guida, Lucio Previtera, Giovanni Di Fabio and Armando Zarrelli*

* Correspondence: zarrelli@unina.it; Tel.: +39-081-674-472

Table S1. ^{1}H, ^{13}C and 2D NMR data of Octocrylene in $CDCl_3$.

Position	Residue	$^{13}C^{a}$	$^{1}H^{a}$, multiplicity (J in Hz)	^{1}H-^{1}H COSY	^{1}H-^{13}C HMBC
1	C	162.90	-		
2	C	104.10	-		
3	C	168.84	-		7.16
4	C	138.33	-		7.41, 7.16
5/9	CH	128.23	7.16, d (7.2)	7.41, 7.38	7.41, 7.38
6/8	CH	129.26	7.41, m	7.38, 7.16	7.38, 7.16
7	CH	130.33	7.38, m	7.41, 7.16	7.41, 7.16
10	C	138.65	-		7.41, 7.16
11/15	CH	128.47	7.16, d (7.2)	7.41, 7.38	7.41, 7.38
12/14	CH	130.21	7.41, m	7.38, 7.16	7.38, 7.16
13	CH	131.55	7.38, m	7.41, 7.16	7.41, 7.16
16	C	116.76	-		
17	CH_2	68.52	4.02, dd (6.5, 5.5)	1.46	1.46, 1.23
18	CH	38.50	1.46, m	4.02, 1.23	4.02, 1.23, 0.83
19	CH_2	30.05	1.23, m	1.46	4.02, 1.46
20	CH_2	28.81	1.23, m	1.23	1.46, 1.23
21	CH_2	22.85	1.23, m	1.23, 0.89	1.23, 0.89
22	CH_3	14.00	0.89, d (7.5)	1.23	1.23
23	CH_2	23.42	1.23, m	1.46, 0.83	4.02, 1.46, 1.23, 0.83
24	CH_3	10.88	0.83, d (7.6)	1.23	1.46, 1.23

[a]Chemical shifts in ppm.

Table S2. ^{1}H, ^{13}C and 2D NMR data of **DP2** in $CDCl_3$.

Position	Residue	$^{13}C^{a}$	$^{1}H^{a}$, multiplicity (J in Hz)	^{1}H-^{1}H COSY	^{1}H-^{13}C HMBC
1	C	194.01	-		7.81
2	C	137.98	-		7.81, 7.49
3/7	CH	130.25	7.81, m	7.59, 7.49	7.59, 7.49
4/6	CH	127.95	7.49, m	7.81, 7.59	7.81, 7.59
5	CH	132.60	7.59, m	7.81, 7.49	7.81, 7.49
8	C	137.98	-		7.81, 7.49
9/13	CH	130.25	7.81, m	7.59, 7.49	7.59, 7.49
10/12	CH	127.95	7.49, m	7.81, 7.59	7.81, 7.59
11	CH	132.60	7.59, m	7.81, 7.49	7.81, 7.49

[a]Chemical shifts in ppm.

Table S3. [1] H, ^{13}C and 2D NMR data of **DP3** in $CDCl_3$.

Position	Residue	$^{13}C^{a}$	$^{1}H^{a}$, multiplicity (J in Hz)	^{1}H-^{1}H COSY	^{1}H-^{13}C HMBC
1	C	172.63	-		
2	CH	57.39	5.06, s		7.34
3	C	138.79	-		5.06, 7.34
4/8	CH	128.51	7.34, m	7.29	5.06, 7.34, 7.29
5/7	CH	128.65	7.34, m	7.29	7.29
6	CH	127.17	7.29, m	7.34	7.34
9	C	138.79	-		7.34
10/14	CH	128.51	7.34,m m	7.29	7.34, 7.29
11/13	CH	128.65	7.34, m	7.29	7.29
12	CH	127.17	7.29, m	7.34	7.34
15	CH_2	67.50	4.10, d (5.5)	1.60	1.60, 1.28
16	CH	38.76	1.60, m	4.10, 1.28	4.10, 1.28
17	CH_2	30.6	1.28, m	1.60	1.60, 1.28
18	CH_2	28.84	1.28, m		1.28
19	CH_2	22.93	1.28, m		1.28, 0.88
20	CH_3	14.01	0.88, t (7.5)	1.28	1.28
21	CH_2	23.76	1.28, m	1.60, 0.88	1.60, 0.88
22	CH_3	10.95	0.88, t (7.5)	1.28	1.60, 1.28

[a]Chemical shifts in ppm.

Table S4. ^{1}H, ^{13}C and 2D NMR data of **DP4** in $CDCl_3$.

Position	Residue	$^{13}C^{a}$	$^{1}H^{a}$, multiplicity (J in Hz)	^{1}H-^{1}H COSY	^{1}H-^{13}C HMBC
1	C	170.13	-		
2	C	81.58	-		7.38, 7.33
3	C	147.32	-		7.38, 7.33
4/8	CH	127.85	7.38, m	7.38	7.38
5/7	CH	128.30	7.38, m	7.38, 7.35	7.38, 7.35
6	CH	127.67	7.35, m	7.38	7.38
9	C	147.32	-		7.33
10/14	CH	127.85	7.38, m	7.38	7.40, 7.33
11/13	CH	128.30	7.38, m	7.38, 7.35	7.40, 7.33
12	CH	127.67	7.35, m	7.38	7.33
15	CH_2	67.08	4.18, d (5.5)	1.55	1.55, 1.24, 1.22
16	CH	36.68	1.55, m	4.18, 1.24, 1.22	4.18, 1.24, 1.22
17	CH_2	30.31	1.24, m<br>1.22, m	1.55, 1.22<br>1.55, 1.24	1.55, 1.24, 1.22
18	CH_2	28.76	1.24, m<br>1.22, m	1.22<br>1.24	1.24, 1.22, 0.83
19	CH_2	22.92	1.24, m<br>1.22, m	1.22, 0.83<br>1.24, 0.83	1.24, 1.22, 0.83
20	CH_3	14.02	0.83, t (7.7)	1.24, 1.22	1.24, 1.22
21	CH_2	23.75	1.24, m<br>1.22, m	1.55, 1.22<br>1.55, 1.24	1.55, 0.81
22	CH_3	10.93	0.81, t (7.7)	1.24, 1.22	1.55, 1.24, 1.22

[a]Chemical shifts in ppm.

Table S5. [1] H, ^{13}C and 2D NMR data of **DP5** in $CDCl_3$.

Position	Residue	$^{13}C^a$	$^1H^a$, multiplicity (J in Hz)	^1H-^1H COSY	^1H-^{13}C HMBC
1	C	161.78	-		
2	C	78.06	-		
3	C	72.96	-		7.55, 7.48
4	C	134.38	-		7.55, 7.43
5/9	CH	127.29	7.55, m	7.44, 7.43	7.44, 7.43
6/8	CH	128.70	7.43, m	7.55, 7.44	7.55, 7.44
7	CH	129.64	7.44, m	7.55, 7.43	7.55, 7.43
10	C	133.69	-		7.48, 7.42
11/15	CH	127.15	7.48, m	7.42, 7.41	7.42
12/14	CH	128.62	7.42, m	7.48, 7.41	7.48, 7.41
13	CH	129.30	7.41, m	7.48, 7.42	7.48, 7.42
16	C	113.78/113.76	-		
17	CH_2	69.81/69.77	4.01, m<br>3.91, m	1.43	1.43, 1.25, 1.22
18	CH	38.51/38.46	1.43, m	4.01, 3.91, 1.25, 1.22	4.01, 3.91, 1.25, 1.22, 0.83
19	CH_2	29.99/29.91	1.22, m	1.43	4.01, 3.91, 1.43, 1.25, 1.22, 0.83
20	CH_2	28.81/28.74	1.22, m	1.25	1.43, 1.25, 1.22, 0.93
21	CH_2	22.86/22.85	1.25, m	1.22, 0.93	1.22, 0.93
22	CH_3	14.03/14.01	0.93, t (7.6)	1.25	1.25, 1.22
23	CH_2	23.36/23.32	1.25, m	1.43, 0.83	4.01, 3.91, 1.43, 0.83
24	CH_3	10.86/10.82	0.83, m (7.6)	1.25	1.43, 1.22

[a]Chemical shifts in ppm.

Table S6. ^{1}H, ^{13}C and 2D NMR data of **DP7** in $CDCl_3$.

Position	Residue	$^{13}C^{a}$	$^{1}H^{a}$, multiplicity (J in Hz)	^{1}H-^{1}H COSY	^{1}H-^{13}C HMBC
1	C	178.16	-		
2	CH	57.05	5.08		7.36
3	C	138.02	-		5.08, 7.36
4/8	CH	128.66	7.36, m	7.31	5.08, 7.36, 7.31
5/7	CH	128.70	7.36, m	7.31	7.36, 7.31
6	CH	127.47	7.31, m	7.36	7.36
9	C	138.02	-		7.36
10/14	CH	128.66	7.36, m	7.31	7.36, 7.31
11/13	CH	128.70	7.36, m	7.31	7.36, 7.31
12	CH	127.47	7.31, m	7.36	7.36

[a]Chemical shifts in ppm.

Table S7. ^{1}H, ^{13}C and 2D NMR data of **DP8** in $CDCl_3$.

Position	Residue	$^{13}C^{a}$	$^{1}H^{a}$, multiplicity (J in Hz)	^{1}H-^{1}H COSY	^{1}H-^{13}C HMBC
1	C	166.53	-		
2	C	105.17	-		
3	C	163.50	-		7.43, 7.15
4	C	138.48	-		
5/9	CH	129.61	7.43, m	7.50, 7.30	7.50, 7.30
6/8	CH	128.14	7.30, m	7.50, 7.43	7.50, 7.43
7	CH	131.29	7.50, m	7.43, 7.30	7.43, 7.30
10	C	138.75	-		
11/15	CH	130.30	7.15, m	7.39	7.39, 7.38
12/14	CH	128.48	7.38, m	7.39, 7.15	7.39, 7.15
13	CH	130.43	7.39, m	7.38, 7.15	7.38, 7.15
16	C	117.65	-		

[a]Chemical shifts in ppm.

Table S8. [1] H, ^{13}C and 2D NMR data of **DP9** in $CDCl_3$.

Position	Residue	$^{13}C^a$	$^1H^a$, multiplicity (J in Hz)	^1H-^1H COSY	^1H-^{13}C HMBC
1	C	170.05	-		
2	C	72.30	-		
3	C	72.69	-		7.54
4	C	138.11	-		7.54, 7.41
5/9	CH	128.61	7.54, m	7.41, 7.33	7.41, 7.33
6/8	CH	129.56	7.41, m	7.54, 7.33	7.54, 7.33
7	CH	129.98	7.33, m	7.54, 7.41	7.54, 7.41
10	C	136.78	-		7.54, 7.35
11/15	CH	128.10	7.54, m	7.35, 7.33	7.35, 7.33
12/14	CH	129.40	7.35, m	7.54, 7.33	7.54, 7.33
13	CH	129.72	7.33, m	7.54, 7.35	7.54, 7.35
16	C	117.85	-		

[a]Chemical shifts in ppm.
